# Audit of the appropriateness of the indication for obstetric sonography in a tertiary facility in Ghana

**DOI:** 10.11604/pamj.2021.40.35.26349

**Published:** 2021-09-14

**Authors:** Emmanuel Kobina Mesi Edzie, Klenam Dzefi-Tettey, Philip Narteh Gorleku, Edmund Kwakye Brakohiapa, Benard Ohene Botwe, Adu Tutu Amankwa, Ewurama Andam Idun, Henry Kusodzi, Abdul Raman Asemah

**Affiliations:** 1 Department of Medical Imaging, School of Medical Sciences, College of Health and Allied Sciences, University of Cape Coast, PMB, Cape Coast, Ghana,; 2 Department of Radiology, Korle Bu Teaching Hospital. P.O. BOX KB 77 Korle Bu, Accra, Ghana,; 3 Department of Radiology, University of Ghana School of Medicine and Dentistry, College of Health Sciences, University of Ghana. P. O. BOX GP 4236, Accra, Ghana,; 4 Department of Radiography, School of Biomedical and Allied Health Sciences, College of Health Sciences, University of Ghana, Accra, Ghana; 5 Department of Radiology, School of Medical Sciences, College of Health Sciences, Kwame Nkrumah University of Science and Technology, Kumasi, Ghana,; 6 Department of Radiology, 37 Military Hospital, Neghelli Barracks Liberation Road 37, Accra, Ghana

**Keywords:** Audit, indications, request forms, obstetric ultrasound, tertiary facility, Ghana

## Abstract

**Introduction:**

the use of ultrasound is one of the most vital tools in the management of pregnancies and contributes significantly in improving maternal and child health. Certain indications in pregnancy, guide the obstetrician as to which obstetric scan deems appropriate. The full realization of the benefits of ultrasound depends on whether it is being used appropriately or not, and hence this study aimed at auditing for the appropriate indications for obstetric ultrasound.

**Methods:**

a review of all request forms for obstetric scan between June 2019 and July 2020 was performed to assess the appropriateness of requests for obstetric ultrasound at the Cape Coast Teaching Hospital. The data obtained was analyzed using SPSS (SPSS Inc. Chicago, IL version 20.0). A Chi-squared test of independence was used to check for statistically significant differences between variables at p ≤ 0.05.

**Results:**

three hundred and fourteen (314) out of the 527 request forms had clinical indications stated. 174 (81.7%) of requests from Cape Coast Teaching Hospital and 39 (18.3%) from other health centers did not indicate patients clinical history/indication on the request forms. Majority 76 (68.5%) of scans in the first trimester were done without indications/history. Only 29 of requests with clinical history were inappropriate.

**Conclusion:**

practitioners should be mindful of adequately completing request forms for obstetric investigations since a large number of practitioners do not state the history/indications for the scans. There should be continuous medical education on the importance of appropriate indication for obstetric ultrasound.

## Introduction

The application of ultrasound imaging in obstetric care has contributed significantly to the improvement of maternal health through the early diagnosis of complications like placenta previa, ectopic pregnancy, and structural problems with the uterus [1]. Aside pregnancy complications, obstetric ultrasonography is a routine practice in radiology, performed to evaluate intrauterine gestation in early pregnancy, fetal anatomy at mid-term or to assess fetal growth at near-term [2]. Obstetric ultrasonography is commonly used to evaluate issues of fetal viability, anomalies and fetal well-being [3]. For instance, an early ultrasound scan (before 13 weeks and 6 days gestation) is normally performed to confirm a viable intrauterine pregnancy whilst second trimester ultrasound examinations are used for fetal anatomic survey, ideally performed between 18-20 weeks and third trimester examinations for detailed fetal growth evaluations, usually performed after 32 weeks of pregnancy [4].

Basically, an obstetric ultrasound examination gives an accurate and safe clinical evaluation of the gravid uterus throughout a woman´s pregnancy [5]. The American College of Radiology (ACR), the American College of Obstetricians and Gynecologists (ACOG), and the American Institute of Ultrasound in Medicine (AIUM) jointly released an updated practice guidelines in 2013 for performing an obstetric ultrasound examinations. For high quality of patients care, these guidelines have described indications and key elements for an obstetrical ultrasound examination. According to these guidelines, “a standard obstetrical ultrasound in the second and third trimester includes determination of amniotic fluid volume, cardiac activity, placental position, fetal number, fetal presentation, fetal biometry, and fetal anomaly scan” [6]. In general, screening for pathological conditions with ultrasound, helps improve maternal and prenatal healthcare due to the radiation free visualization of the fetus, uterus and placenta [7]. These applications show the appropriate uses of ultrasound technology, since they provide a clear and early diagnosis of potential problems [8]. The challenge with ultrasonography is where useful clinical information from the requesting practitioners are missing, which may affect the accuracy of the ultrasound procedure as well as the interpretation of results [9], thereby creating the possibility of patient mismanagement. In a study conducted in Norway, majority of obstetricians reported that even in the absence of medical indications, pregnant women will always expect to undergo an ultrasound examination [10]. Most sonographers on the other hand, particularly in private diagnostic facilities, do not bother about the clinical indications because of the economic gains derived from the procedure [11]. Even though there is no conclusive evidence of harm in human studies, if used imprudently, diagnostic ultrasound could be capable of producing harmful effects [12].

Ultrasound is arguably, the most commonly used diagnostic procedure in obstetrics [13]. In our setting, a recent study conducted by Edzie et al. to assess the imaging modalities available in radiological practices in Ghana, discovered that, Digital Ultrasound was the commonest among all imaging modalities available [14]. Therefore, it is important to adhere to the protocols for requesting an obstetric ultrasound and maintain vigilance to ensure the continued safe use of ultrasound and this is exactly what “as low as reasonably achievable” (ALARA) recommends [15]. However, it is assumed that the subjective opinions of midwives and obstetricians will influence their requests for ultrasound examination leading to inappropriate requests. In this study, we audited for the appropriateness of indications for obstetric ultrasound in a tertiary facility in Ghana. Most professional organizations have laid down guidelines for professional ultrasound practice in obstetrics. The present study compared the findings to the ACR-AIUM-ACOG composite practice guidelines for the use of obstetrical ultrasound as the yardstick for the appropriateness of requests.

## Methods

### Study design

This retrospective study reviewed all request forms of clients who had an obstetric scan at the imaging center of the Cape Coast Teaching Hospital (CCTH) between June 2019 and July 2020. The facility is a public tertiary facility that receives referrals from all tiers of health care delivery centers in the region. All the scans were done using a Toshiba ultrasound machine (Nemio XG Toshiba American Medical system, Inc. Tustin California) fitted with curvilinear transducers with frequency of 2.5MHz. Three consultant radiologists of at least 15 years experience in obstetric ultrasonography performed all the scans.

### Data collection

The reports for all the requests were retrieved from the electronic records; Picture Archiving and Communications System (PACS) by the radiologists after permission from the hospital authorities. The clinical history and age of the gravid women were recorded as indicated on the request forms, and the maturity of pregnancies were recorded. Forms were only included if their corresponding scan report were retrievable, or otherwise were excluded. All request forms were checked to differentiate requests from CCTH and those from outside CCTH. All requests were classified as either appropriate or inappropriate. Two radiologists who are both authors of this study independently evaluated the appropriateness of each request by comparing the clinical details or history provided on the request forms to the composite American College of Radiology (ACR) - American Institute of Ultrasound in Medicine (AIUM) - American College of Obstetricians and Gynecologists (ACOG) practice guidelines for the performance of obstetrical ultrasound [6]. Two criteria of appropriateness were considered. First, all request forms without history/indication were considered as inappropriate and those with history/indication were considered as appropriate. For request forms with clinical history/indication, a second measure of appropriateness was used. A request was classified as appropriate if the indications of the scans requested and the maturity of pregnancies conform to the ACR- AIUM - ACOG guidelines.

### Statistical analysis

Data obtained (clinical history/indication, gestational age, origin of requests, and demographics) were entered in SPSS (SPSS Inc. Chicago, IL version 20.0) software for windows and analyzed using frequencies, percentages, and presented in appropriate tables and charts. We compared the appropriateness of request forms (with or without history) and scan indications from CCTH and outside CCTH using Chi-square. A p-value ≤ 0.05 was considered statistically significant in all inferential analyses.

### Ethical consideration

The study was approved by the Ethical Review Board of Cape Coast Teaching Hospital with clearance number CCTHERC/EC/2020/057. Anonymity and confidentiality were maintained throughout the study.

## Results

A total of 527 request forms were retrieved and reviewed for this study. The mean age was 29.96±5.070 ranging from 14 to 50 years. Majority (45.5%) of the scans were done in the third trimester (Table 1). Overall, 314 out of the 527 request forms had clinical indications stated. The number of requests that were appropriate was 295 (93.3%) largely from CCTH practitioners. However, a significant number 174 (81.7%; p< 0.001) of the request forms without clinical history/indication were from CCTH. Also, out of the 58 request forms from other health centers, 39 of them did not write the history of the patient. For request forms with clinical history, only a few 29 (9.2%) were inappropriate. Comparative analysis using Chi-squared test showed that inappropriate requests from the other health centers were significantly higher than requests from practitioners within CCTH (P < 0.001) (Table 1 and Table 2).

**Table 1:** demographic characteristics

Variable	Count (%)
**Age**	
Min.	14
Max.	50
Mean(SD)	29.96(5.070)
**Trimester**	
First (1-12 weeks)	111(21.1%)
Second (13-26 weeks)	176(33.4%)
Third (27weeks to term)	240(45.5%)
**Analysis of request forms**	
Presence of history/indication	314(59.6%)
No history/indication	213(40.4%)
Patient name	527(100%)
Patient age	527(100%)
**Origin of Request**	
CCTH	469(89.0%)
Outside CCTH	58(11.0%)
**Appropriateness of scan indication**	
Appropriate	286(90.8%)
Inappropriate	29(9.2%)

**Table 2:** appropriateness of scan requests from practitioners

Variable	CCTH	Outside CCTH	P-value
**Appropriateness of request form**			
Appropriate	295(93.9%)	19(6.1%)	P < 0.001*
Inappropriate	174(81.7%)	39(18.3%)	
**Appropriateness by international guidelines**			
Appropriate	276(96.5%)	10(3.5%)	P < 0.001*
Inappropriate	19(65.5%)	10(34.5%)	

* Statistically significant

**Figure 1:**
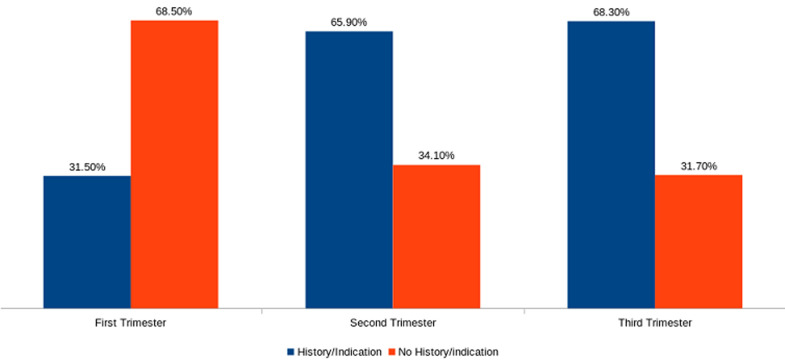
proportion of request forms with/without history/indication in the various trimesters

**Figure 2:**
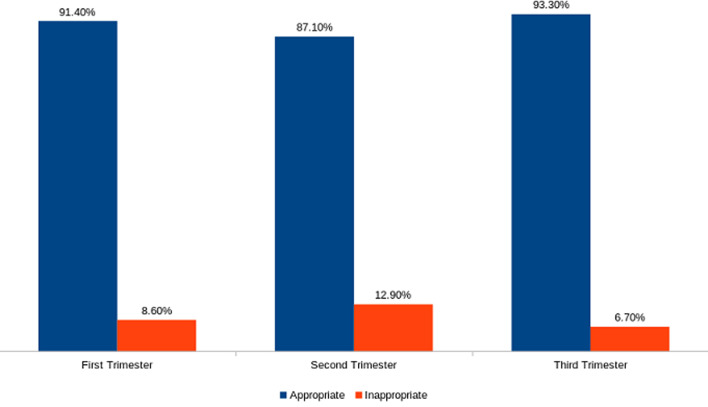
appropriateness of clinical indications in the various trimesters compared with ACR-AIUM-ACOG guidelines

A total of 111, 176 and 240 scans were recorded in the first, second and third trimester respectively. Majority 76 (68.5%) of scans in the first trimester were done without indications. In the second and third trimesters, most practitioners stated indications for the scan [116 (65.9%) in the second trimester and 164 (68.3%) in the third trimester] (Table 1 and Figure 1). Majority of the obstetric request forms with the appropriate indications were high across all the trimesters, with the third trimester scans having the lowest frequency (6.7%) of inappropriate requests (Figure 2). Fetal anatomy was the commonest indication in the second trimester 85(73.3%) and requests for fetal measurements, lie, presentation, liquor and placental assessment were the common indications in the third trimester 107(65.2%) (Table 3). Out of the 314 request forms with scan indications, fetal anomaly and pregnancy dating were the only two indications that were requested outside the required/ideal gestational period (Table 4).

**Table 3:** scan indications for the various trimesters

Indications	First trimester	Second trimester	Third trimester
High Risk Pregnancy	2(5.7%)	9(7.8%)	12(7.3%)
Fetal Viability	5(14.3%)	2(1.7%)	2(1.2%)
Fetal Anomaly	3(8.6%)	85(73.3%)	11(6.7%)
Confirmation of pregnancy	8(22.9%)	2(1.7%)	1(0.6%)
Fetal Measurement, Lie, Presentation, Liquor Assessment and Placental Assessment	6(17.1%)	3(2.6%)	107(65.2%)
Bleeding and Abdominal Pain	10(28.6%)	4(3.4%)	3(1.8%)
Amniotic Fluid Disorders	0(0.0%)	5(4.3%)	6(3.7%)
Anemia in Pregnancy	0(0.0%)	1(0.9%)	3(1.8%)
Dating of Pregnancy	1(2.9%)	5(4.3%)	0(0.0%)
Placenta Previa	0(0.0%)	0(0.0%)	2(1.2%)
Fetal Macrosomia	0(0.0%)	0(0.0%)	1(0.6%)
Trial of Labour after Cesarean	0(0.0%)	0(0.0%)	5(3.0%)
Biophysical Profile for Post Date	0(0.0%)	0(0.0%)	11(6.7%)

**Table 4:** inappropriate indications for scan by trimester

Trimester	Indications	Count (%)	Maturity
First Trimester	Fetal Anomaly	3(100%)	5W+0D
			10W+6D
			12W+3D
Second Trimester	Fetal Anomaly	14(93.3%)	24W+2D
			25W+0D
			25W+6D
			25W+4D
			26W+2D
			25W+2D
	Dating of Pregnancy	1(6.7%)	26W+0D
Third Trimester	Fetal Anomaly	11(100%)	27W+2D
			32W+5D
			33W+2D
			35W+0D
			38W+3D
			32W+1D
			36W+0D
			28W+0D
			35W+0D

## Discussion

The advent of ultrasound in medical practice has had a significant influence on patient management due to its accuracy in diagnosing medical conditions [5]. The clinical application of ultrasound does not involve the use of ionising radiation, hence poses insignificant risk to the developing fetus [12]. Despite the clinical benefits of ultrasound and minimal risk, there is the need to check for the appropriate use of ultrasound, especially for obstetrical reasons. To the best of our knowledge, this study is the first of its kind conducted in a tertiary facility in our setting and provides important insights into the utilization of obstetric ultrasound. The ACR, ACOG and AIUM are all specialized bodies dedicated to advancing the safe and effective use of ultrasound in medicine through professional and public education [16, 17]. These practice guidelines have been developed for use by practitioners performing obstetric ultrasound. According to the ACR, ACOG-AIUM, obstetric ultrasound examination should be performed only when there is a valid medical reason [6]. However, these guidelines are not intended to establish a legal standard of care, but for the purposes of providing high quality ultrasound examination for the betterment of patient care [6].

We found that 40.4% of the request forms had no clinical history/indication (Table 1). Several studies have shown flaws of practitioners in filling of radiological request forms [18, 19]. An analysis of request forms from a previous study showed that a significant proportion of requests forms had no clinical information [20]. It was established that clinicians who request an imaging examination frequently experience several difficulties in getting the clinical history of the patient, mainly due to little/no information described in clinical records of patients and delay in laboratory results [20]. Request forms serve as media of communication between clinicians and diagnostic service providers. Some clinicians underestimate the importance of request forms and hence either do not provide them at all or provide inadequate history/indications when making a radiological request. This may result in medical errors or delay in instituting appropriate treatment [21]. In this study, except for not indicating the clinical history on request forms, the patients´ names and ages were provided on all request forms. Even though this was not an objective for this study, we however found that practitioners from Ghana are doing well compared to other practitioners from other African countries. Similar studies in Ghana on analysis of request forms showed a 99% completion rate of request forms in terms of the patient´s name and age [22]. In a study conducted in South Africa and Nigeria, a parameter like patient age was reported to be filled in as low as 29% and 68% of request forms respectively [23, 24]. The American College of Radiologists (ACR) stipulates that for a better understanding of the patient´s condition, all forms should be adequately completed [18] to aid in the proper management of patients. Likewise, the ACR-ACOG-AIUM, practice guidelines require that “a written request for an obstetrical ultrasound examination should provide sufficient information to demonstrate the medical necessity of the examination” [6].

For request forms that had clinical history/indications, only a few (29 out of 314) were inappropriately requested based on the ACR-ACOG-AIUM guidelines (Table 1). These inappropriate requests were made for the purpose of dating pregnancy with maturity of 26W + 0 D gestation for 1 request form, fetal anomaly for pregnancies with maturity of < 13W gestation in 3 of the request forms and maturity > 22W gestation in 25 of the request forms (Table 4). Pregnancy dating is accurately determined in first-trimester ultrasound (ultrasound before 13 weeks and 6/7 days) and second trimester ultrasound (before 22 0/7 weeks) since an error of dating advanced pregnancy by ultrasound can be significantly enormous [25]. Third-trimester ultrasound (beyond 28 0/7 weeks) is the most inaccurate method for pregnancy dating with an accuracy of +/- 21 to 30 days [26]. In the first trimester, an average of three crown-rump length measurement is used to improve accuracy. When crown-rump length exceeds 84 mm (approximately 14 weeks and 0/7 days), the accuracy decreases, and full fetal biometry is used to approximate gestational age [25]. One major concern with third trimester ultrasound dating is underestimating the gestational age of a growth-restricted fetus [25]. Decisions on pregnancy management using a third-trimester ultrasound alone can be difficult for this reason.

Using ultrasound to assess fetal anomaly is now a routine obstetric practice because of the important component it has in prenatal care [27]. According to the ACR, AIUM, ACOG practice guidelines, second trimester (weeks 18 to 22) is the ideal time for screening for structural defects in pregnancy due to a proper visualization of structures at this period [28]. The majority of fetal anomalies can be diagnosed in late first or early second trimester of pregnancy [28]. Though early first trimester ultrasound can aid in evaluating fetal anatomy, in most instances it is not technically feasible for normal pregnancies [29] and hence inappropriate. The second trimester scan has a higher rate (ranging from 21% to 85%) of detecting major structural anomalies compared to the first trimester scan (13% to 43.6%) [29-32]. Initiating anatomy scans in the first trimester will necessitate an additional ultrasound visit, an extra cost and may be time-wasting [33]. It is likely that for the first trimester anatomic survey, the unique features of first trimester anatomy may be misdiagnosed as a fetal anomaly [29]. Until second trimester, some normal fetal structures (e.g. the cerebellar vermis) are not fully formed and a reassurance to rule out abnormalities in these structures in the first trimester is difficult [28]. In the same light, detecting fetal anomalies in the third trimester is technically more challenging due to fetal growth, poor imaging with static ultrasound and decreased quantities of amniotic fluid [34]. Also, depending on the fetal position, examination of the fetal face, sacrum and extremities may not be possible [35]. Scans performed beyond 22 weeks gestation may limit the ability to seek pregnancy termination. A limitation for this study was that, we only focused on the ACR-AIUM-ACOG practice guidelines as the only measure of appropriateness. The practice guidelines for the performance of obstetric ultrasound differ from one organization to the other.

## Conclusion

This study shows that a large number of practitioners who request for obstetric scans do not state the clinical history/indication of the patients on the request forms. This may affect the quality of service rendered and in effect will affect clinical decisions and management of patients. There is a need to continuously remind practitioners of the importance of adequately completing request forms for investigations as this may be the only means of communication between the clinician and the imaging practitioners. Relating to all ultrasound examinations, sufficient clinical details are required to ensure the right examination is performed. Also, most of the practitioners´ requests were appropriate for the scan indication. Though this finding is laudable, as far as the patient´s health is concerned, the authors suggest that there should be continuous medical education on the importance of appropriate indication for obstetric ultrasound. An understanding of the various indications for first, second and third trimester ultrasound is important to ensure that ultrasound is used only when it is appropriate.

### What is known about this topic

- Adequate and relevant history must be provided when making an obstetric ultrasound scan request;
- The type of examination done and examination findings must conform to standard guidelines.

### What this study adds

- We found that a large number of obstetric practitioners did not provide clinical history/indication on request form;
- Most of the indications for the obstetric scans were appropriate indicative of adherence of practitioners to international practice guidelines when making their requests.
